# Supplementary figures and images for: RNA cis-regulators are important for Streptococcus pneumoniae in vivo success
Source: PLoS Genet. 2024 Mar 5;20(3):e1011188. doi: 10.1371/journal.pgen.1011188 (PMC10942264; doi:10.1371/journal.pgen.1011188)

Figure S1

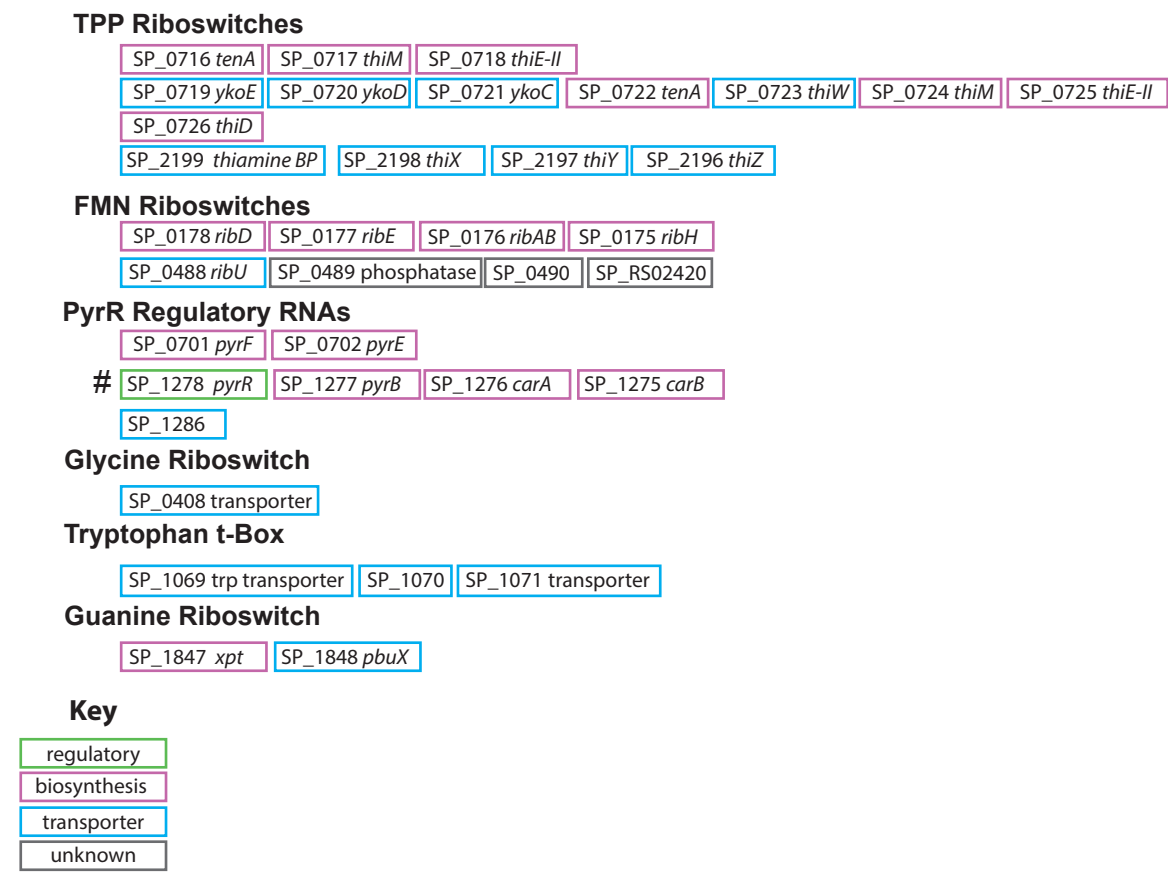

Supplement: S1 Fig — Locus tags and gene names provided where available. Gene function (transport, biosynthesis, regulatory, unknown) indicated by the color of the box. Operon structure determined in a previous sequencing study [64]. #This regulator was assessed as part of a previously published study [64]. (PDF) [file pgen.1011188.s008.pdf]

Figure S3 pg 1

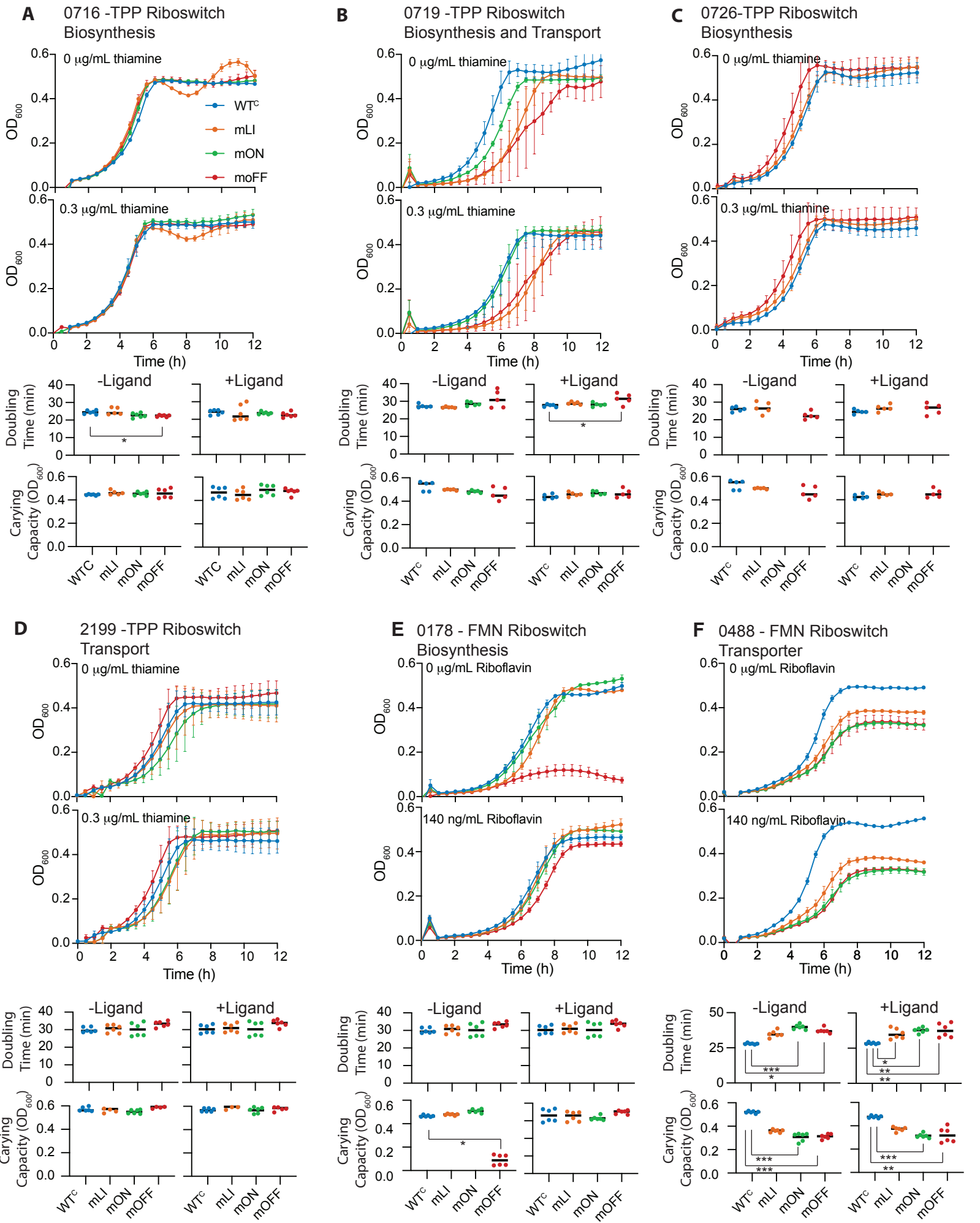

**Figure S3** pg 2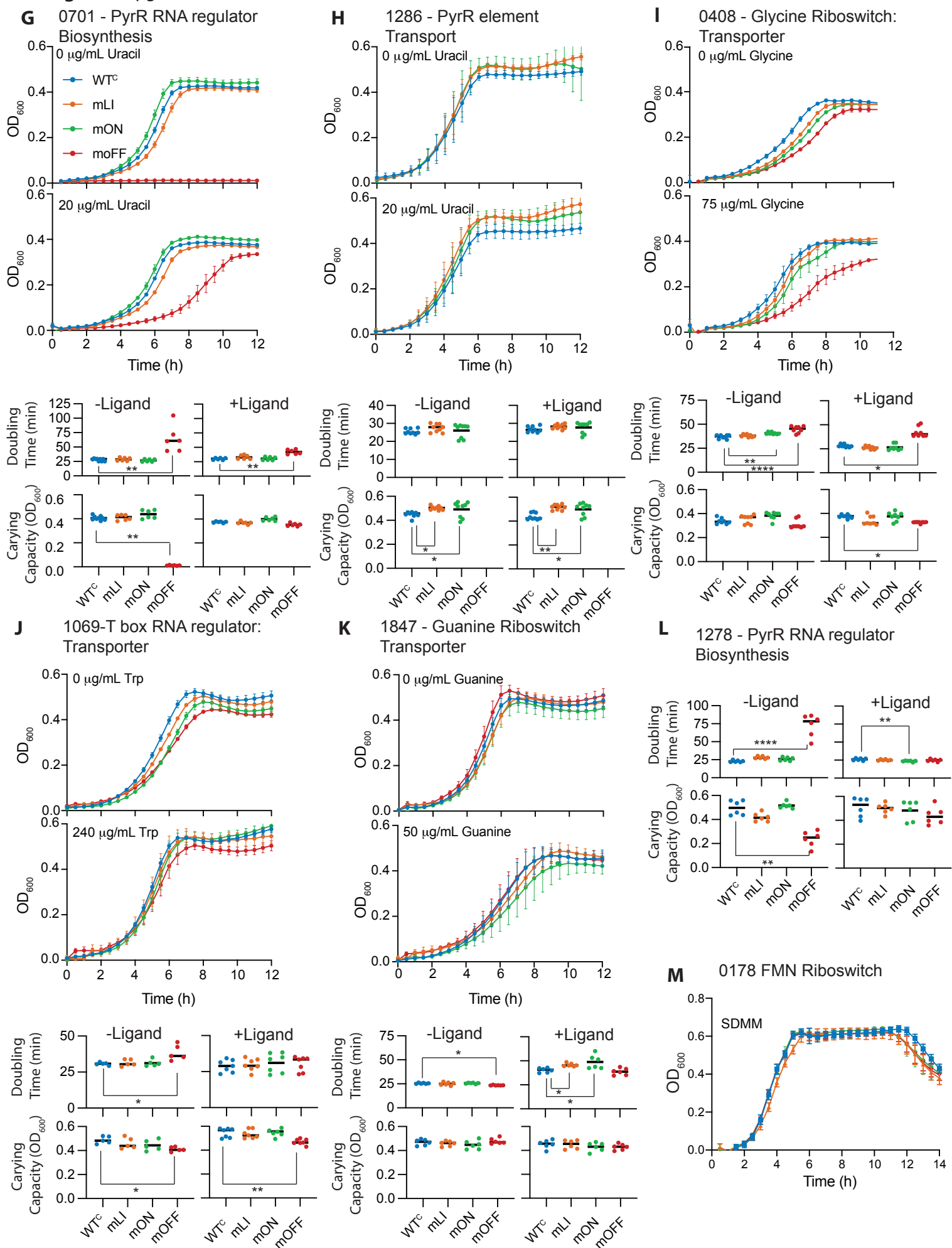

Supplement: S3 Fig — Growth curves in a complete synthetic medium (CDM) in the presence and absence of ligand. All curves represent at least two biological replicates. Error bars represent standard error across all replicates (n = 5–9). Doubling time and carrying capacity measurements extracted from each individual curve under each condition are indicated on graphs below. In each condition mutants were compared to the WTC via Kruskal-Wallis test followed by Dunn’s test of multiple comparisons, those displaying significant changes are indicated (*p<0.05, **p<0.01, ***p<0.001, ****p<0.0001) (A) 0716_TPP mutants grown in the presence and absence of thiamine. (B) 0719_TPP mutants grown in the presence and absence of thiamine. (C) 0726_TPP mutants grown in the presence and absence of thiamine. (D) 2199_TPP mutants grown in the presence and absence of thiamine. (E) 0178_FMN mutants grown in the presence and absence of riboflavin#. (F) 0488_FMN mutants grown in the presence and absence of riboflavin#. (G) 0701_pyrR regulator mutants grown in the presence and absence of uracil#. (H) 1286_pyrR mutants grown in the presence and absence of uracil#. (I) 0408_Glycine mutants grown in the presence and absence glycine. (J) 1069_TrpT-box mutants grown in the presence and absence tryptophan. (K) 1847_Guanine mutants grown in the presence and absence of guanine. (L) Parameters extracted from previously published growth curves for the 1278_pyrR mutants [64] (M) Growth curves in a semi-defined minimal media (SDMM) for 0178_FMN_WTC and mutants showing autolysis phase of characteristic of S. pneumoniae growth in richer medium. #These growth curves are also shown in Figs 6 or 7, but repeated here for accessibility to the entire data set. All numeric data points in S3 Data. (PDF) [file pgen.1011188.s010.pdf]

Figure S4

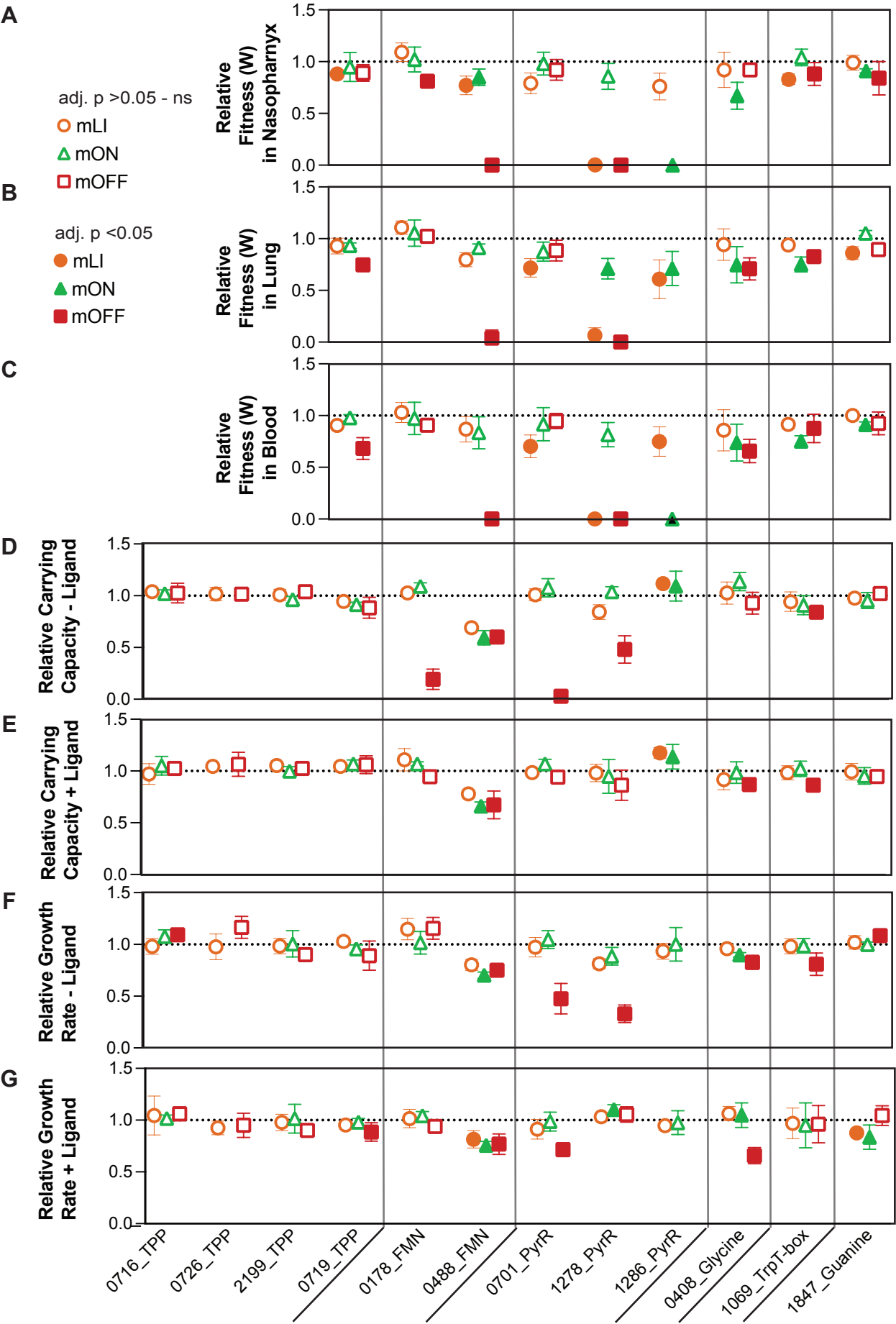

Supplement: S4 Fig — The fitness mean of each individual strain is displayed for nasopharynx colonization (A), lung infection (B), and transition to blood models (C). Orange circles = mLI (Ligand Insensitive), green triangle = mON (constitutively active gene expression), and red square = mOFF (repressed gene expression), error bars correspond to the standard deviation (S4 Table). Filled points correspond to strains with a statistically significant change (p. adj. <0.05) from the in vitro competition (rich medium) control (Kruskal-Wallis test followed by Dunn’s test of multiple comparisons, adj. p<0.05). Open points are not significantly distinct from the in vitro control competition. The black-filled point (1286_pyrR_mON) corresponds to an environment under which none of the mutant strain was recovered despite repeated attempts, indicating a fitness close to 0. Graphs representing individual mouse competitions for each strain are found in S5 Fig. Relative carrying capacity (D,E) and relative growth rate (F,G) for mutant strains compared to the WTC strain. Points represent the mean of 5–9 replicates, and the error bars represent the standard deviation (S3 Table). Some error bars are smaller than the size of the point and therefore not visible. Open points are not statistically significantly different from the WTC strain. Colored points represent values that are statistically significant from the WTC strain (Kruskal-Wallis test followed by Dunn’s multiple comparisons test, adj. p <0.05, S4 Fig and S3 Table). Vertical lines separate groups of cis-acting regulators interacting with the same ligand. All numeric data points in S2 Data. (PDF) [file pgen.1011188.s011.pdf]

**A**

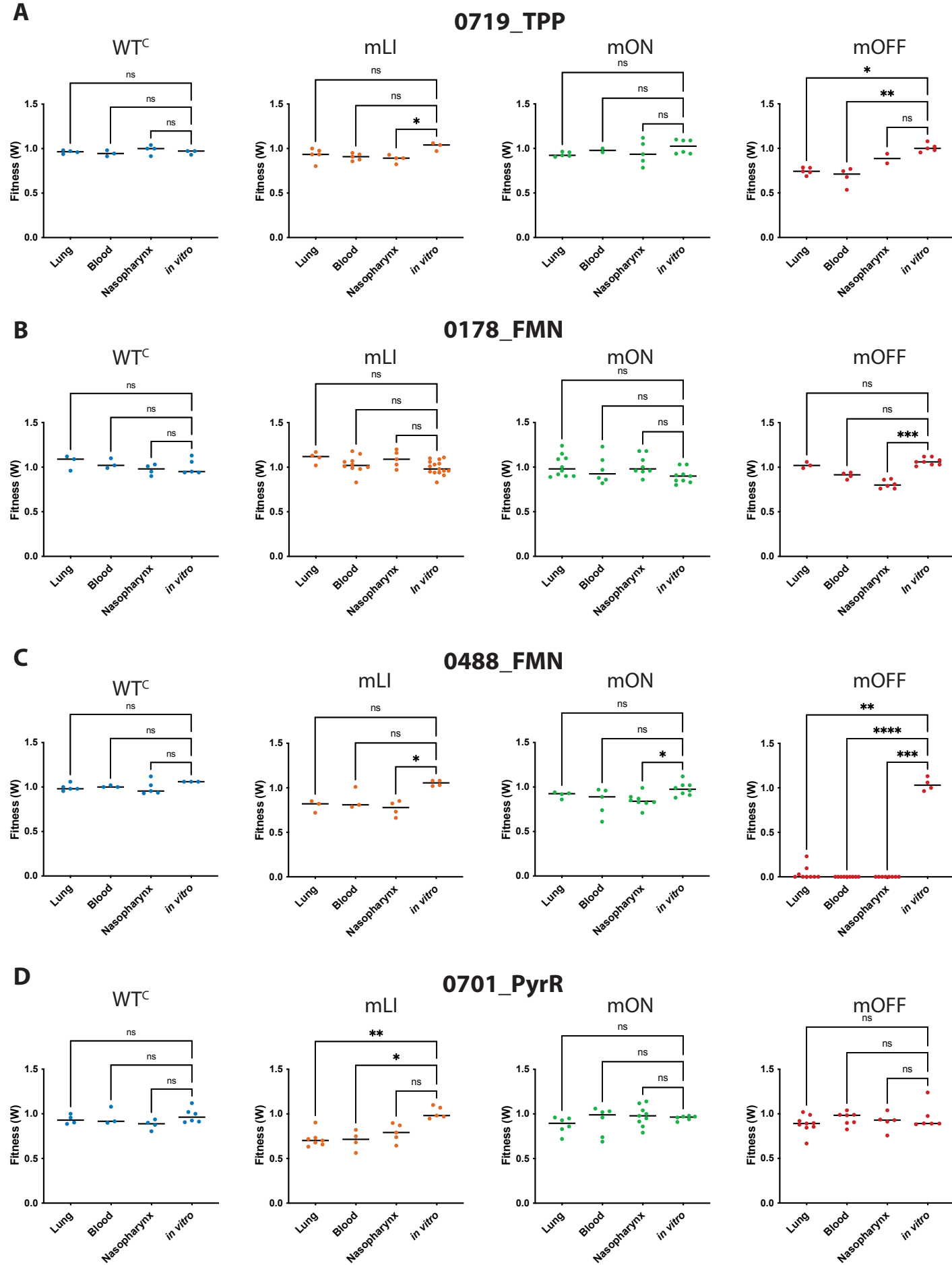

**Figure S5 pg 2**

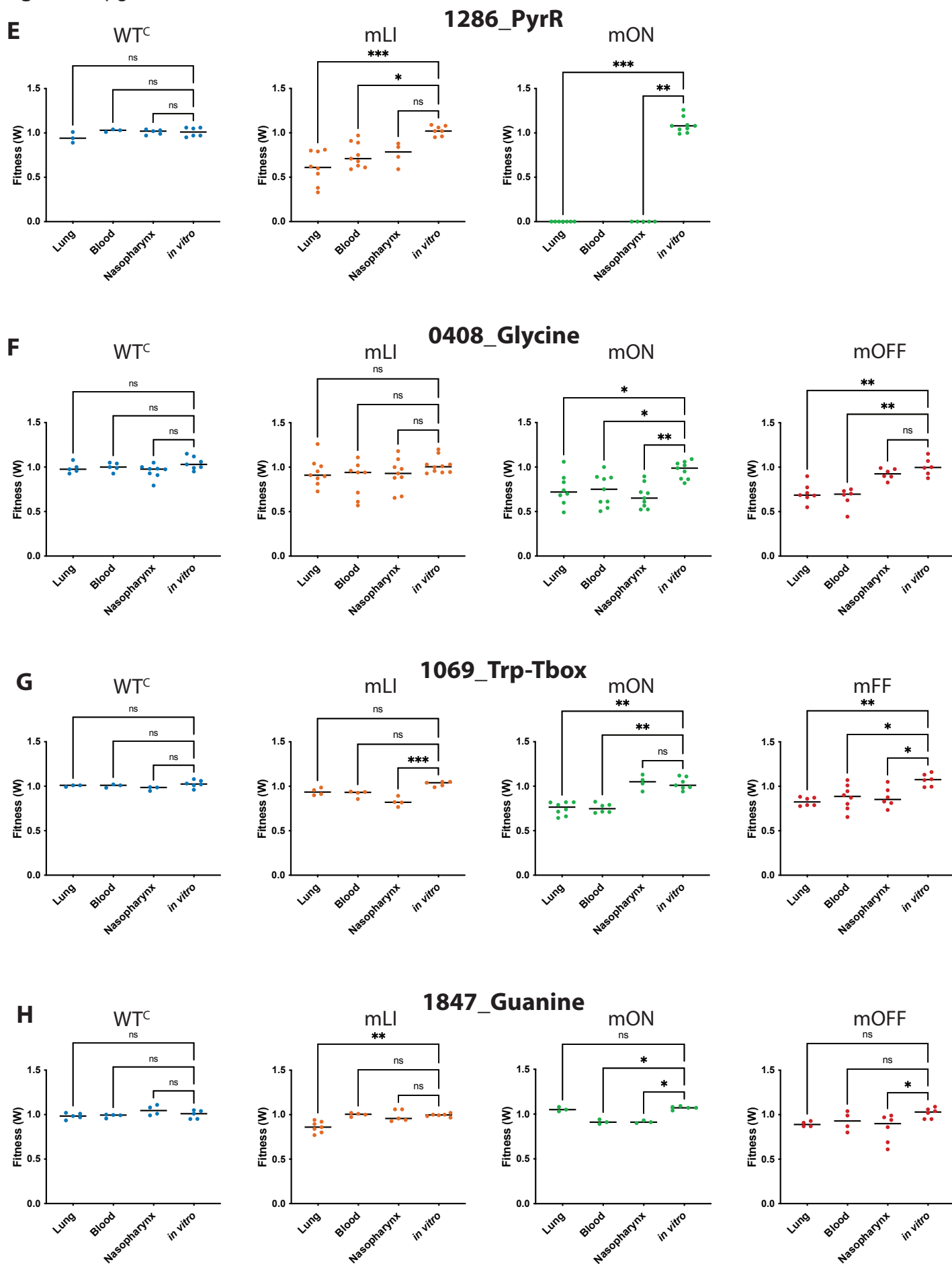

Supplement: S5 Fig — Each data point consists of a fitness value determined from a single mouse infection, or in vitro competition in rich medium. Statistical difference from the control experiment determined using the Kruskal-Wallis test followed by Dunn’s test of multiple comparisons. (* = adj. p<0.05, ** adj. p<0.01, *** p < .001, **** adj. p < .0001). (A) 0719_TPP WTC, mLI, mON and mOFF. (B) #0178_FMN WTC, mLI, mON and mOFF. (C) #0488_FMN WTC, mLI, mON and mOFF. (D) #0701_pyrR WTC, mLI, mON and mOFF. (E) #1286_pyrR WTC, mLI, and mON (1286_pyrR_mOFF mutant not successfully constructed). (F) 0408_Glycine WTC, mLI, mON and mOFF. (G) 1069_Trp-Tbox WTC, mLI, mON and mOFF. (H) 1847_Guanine WTC, mLI, mON and mOFF. #These graphs are also shown in Figs 6 or 7, but repeated here for accessibility to the entire data set. All numeric data points in S2 Data. (PDF) [file pgen.1011188.s012.pdf]

Figure S6

A

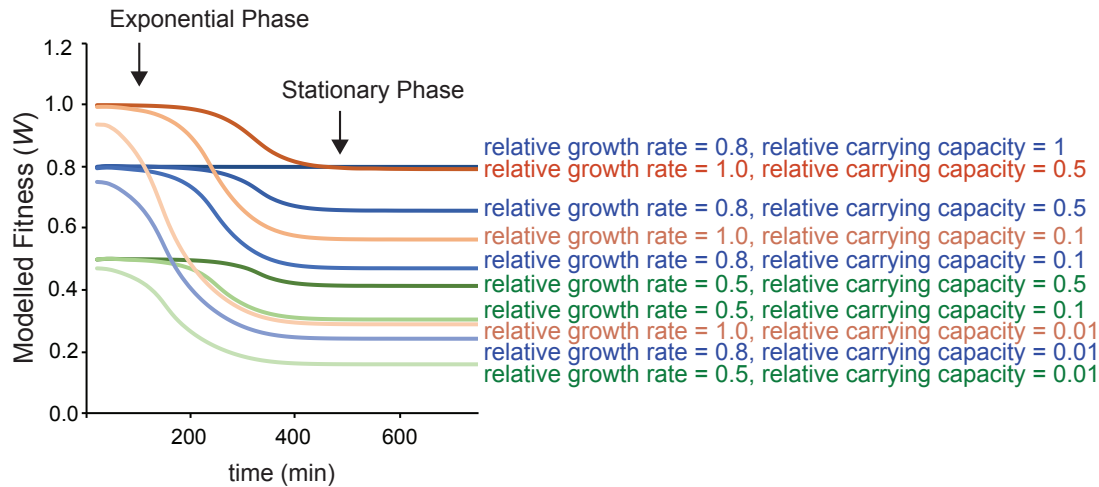

B

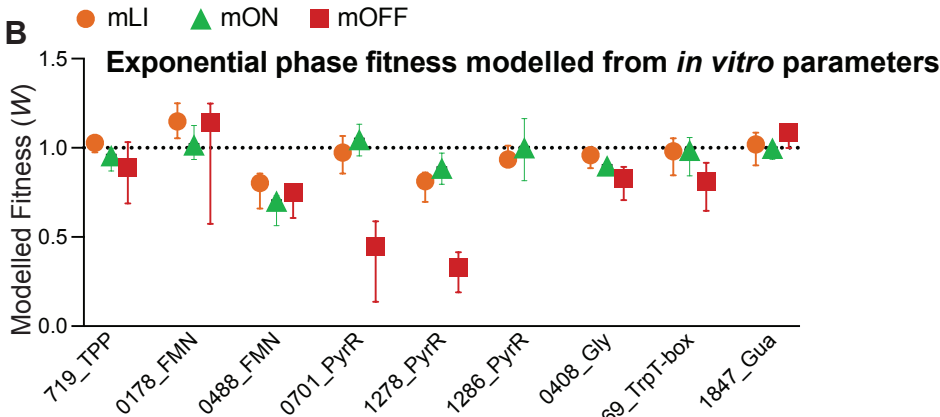

C

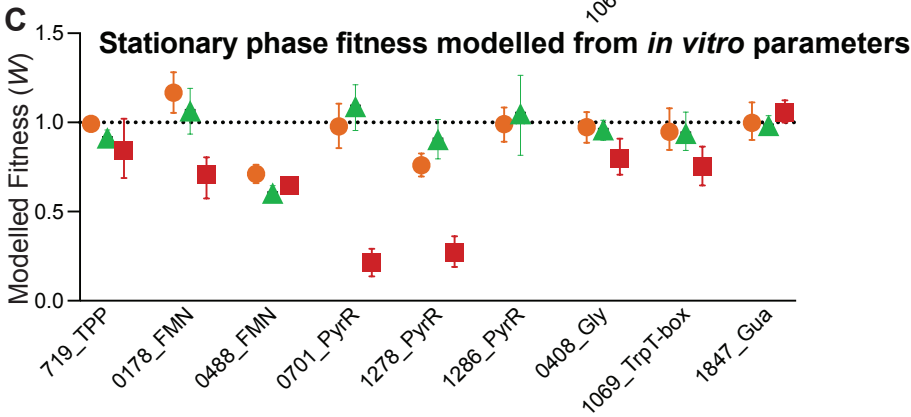

D

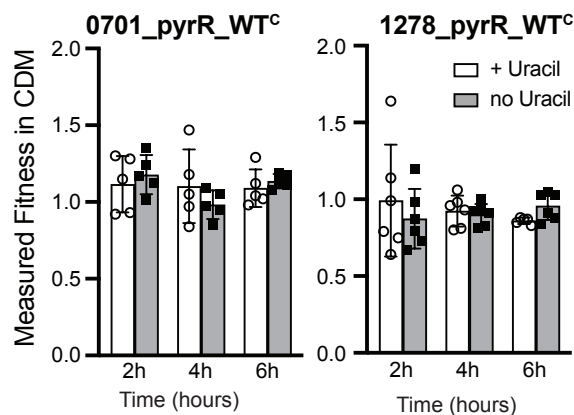

Supplement: S6 Fig — (A) Fitness modelled using a variety of parameter sets as indicated to demonstrate sensitivity of the modelled fitness to parameters. (B) Modelled fitness during exponential phase (120 minutes) and (C) stationary phase (500 minutes) for all RNA regulator mutants based on their relative growth rate and carrying capacity in CDM lacking the target nutrient as reported on S3 Table. Modelled fitness calculated based on a co-culture model with the growth rate of the reference strain ra = 0.0252 and carrying capacity Ka = 0.462. Growth rate and carrying capacity of test strains are scaled by the relative growth rate and carrying capacity such that rb = ra*relative growth rate, and Kb = Ka*relative carrying capacity on S3 Table. Horizontal line drawn at fitness W = 1 for visual reference. Error was estimated by repeating calculations with values of ra and Ka adjusted by the standard deviations of the mean reported on S3 Table. (D) Negative control in vitro competitions conducted between S. pneumoniae TIGR4 and 0701_pyrR_WTC or 1278_pyrR_WTC show no significant change in fitness. Bars represent mean fitness and error bars standard deviation for individual biological replicates shown as points. All numeric data points in S2 Data. (PDF) [file pgen.1011188.s013.pdf]

**Figure S7**

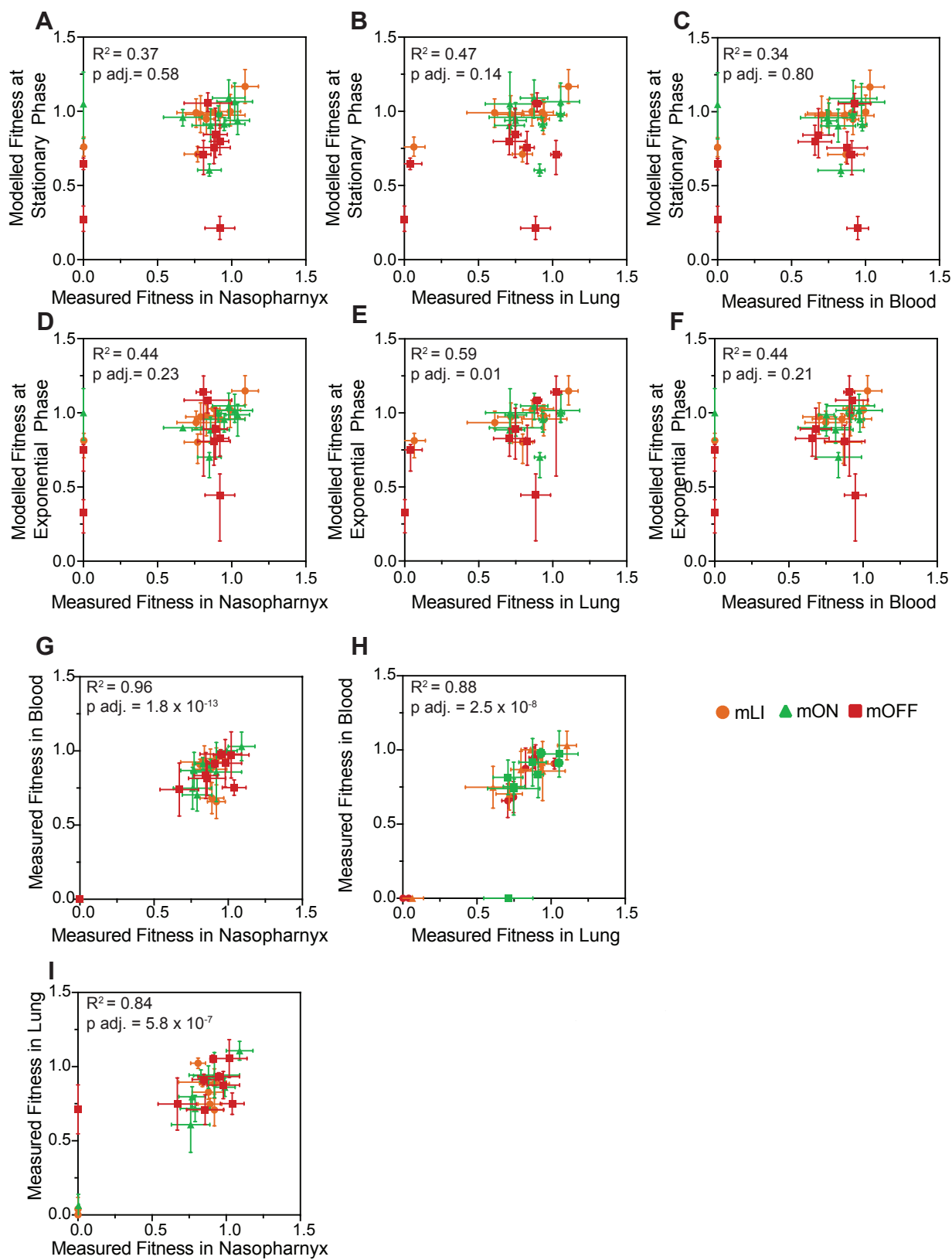

Supplement: S7 Fig — (A-C) Modelled stationary phase fitness vs. mean measured fitness in the nasopharynx, lung, and blood. (D-F) Modelled exponential phase fitness vs. mean measured fitness in the nasopharynx, lung, and blood. (G-H) Comparison of the three different in vivo environments shows significant correlation in fitness values. Pearson’s correlation coefficient and Bonferroni adjusted p-value (N = 9) are reported for each comparison. Error bars correspond to error bars reported in S4A–S4C, and S6B, S6C Figs. All numeric data points in S2 Data. (PDF) [file pgen.1011188.s014.pdf]
