# Supplementary material for: RNA cis-regulators are important for Streptococcus pneumoniae in vivo success
Source: PLoS Genet. 2024 Mar 5;20(3):e1011188. doi: 10.1371/journal.pgen.1011188 (PMC10942264; doi:10.1371/journal.pgen.1011188)

Figure S2 pg 1

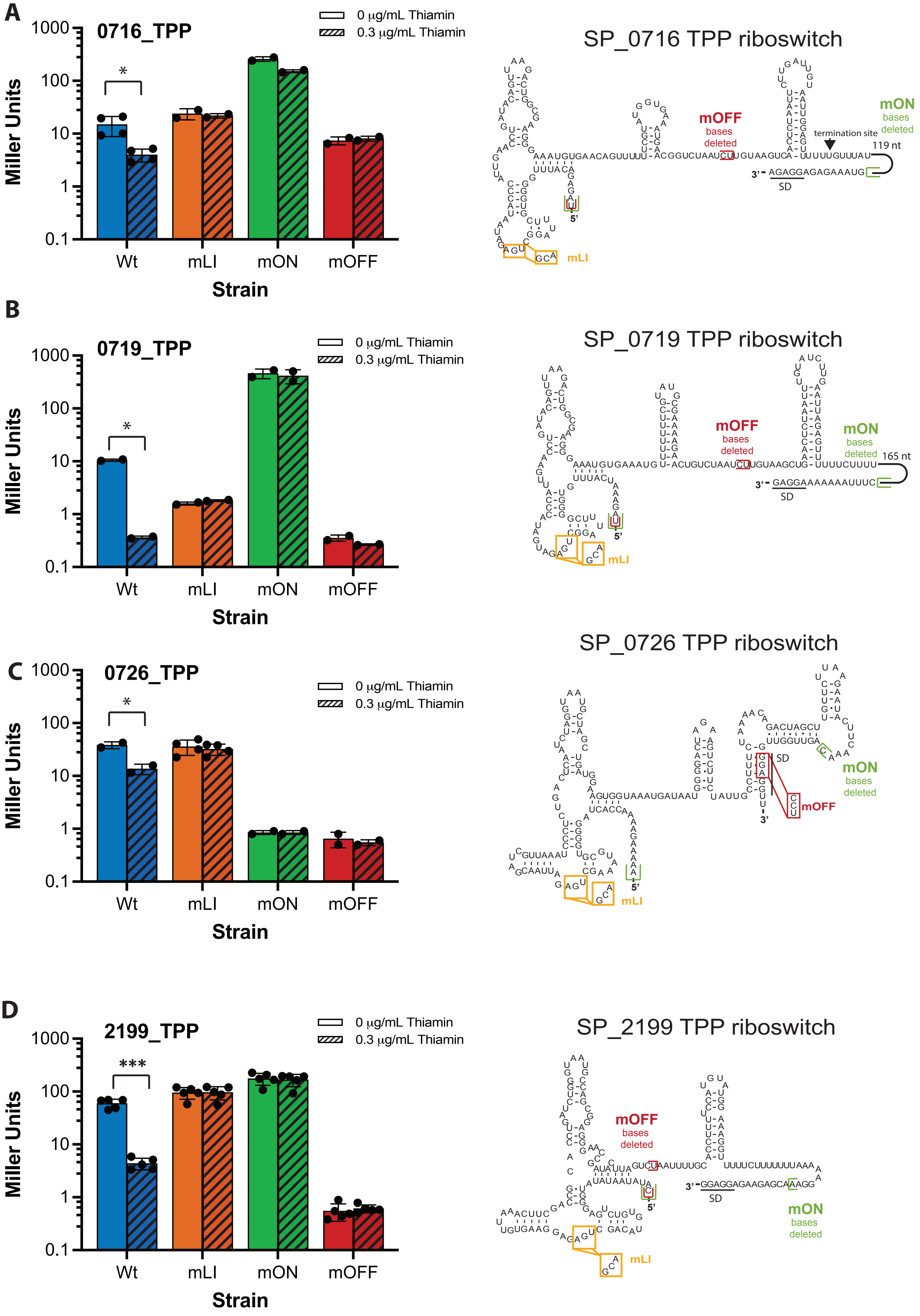

Figure S2 pg 2

E

SP_0178 FMN riboswitch

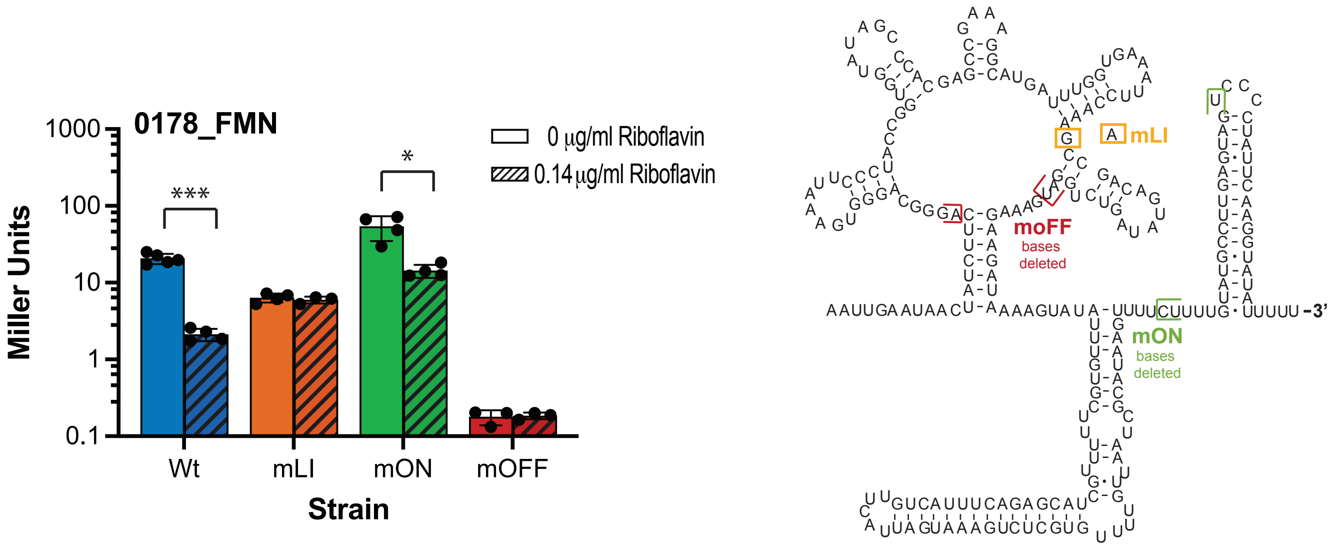

F

SP_488 FMN riboswitch

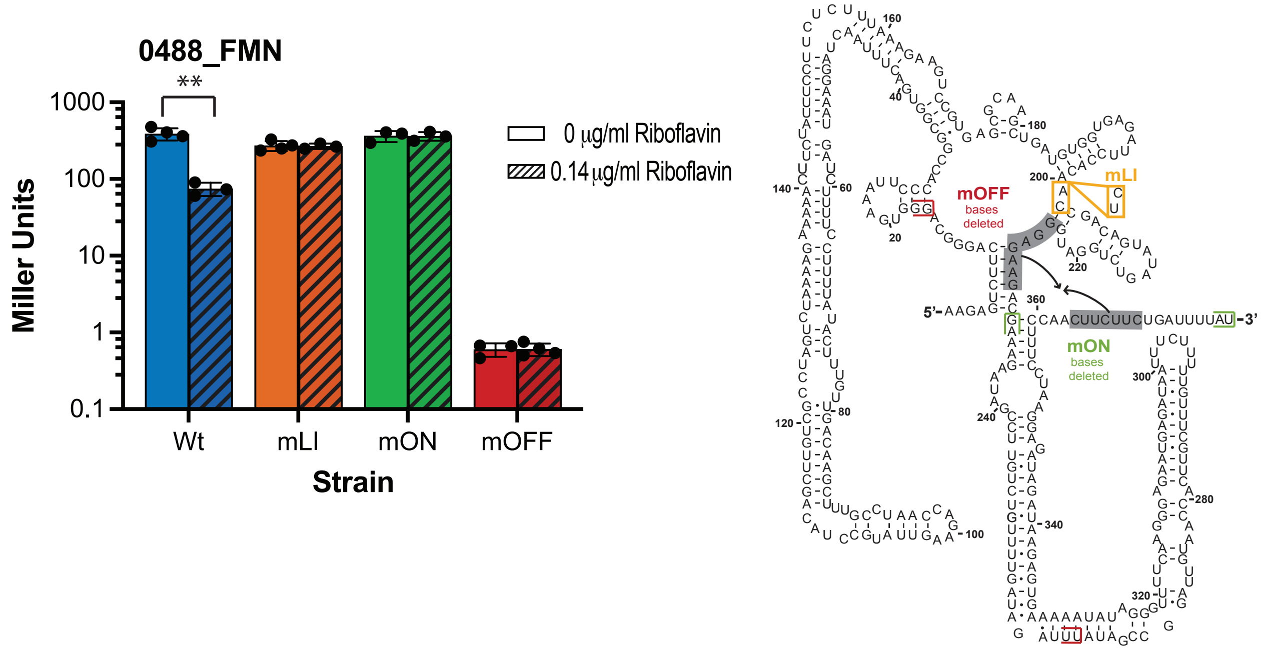

Figure S2 pg 3

G

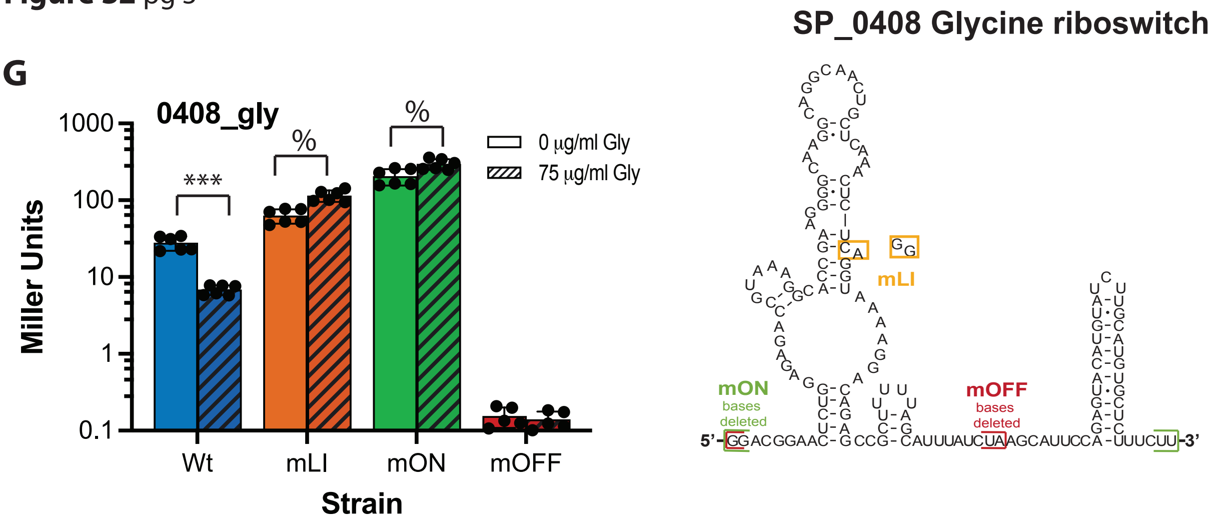

H

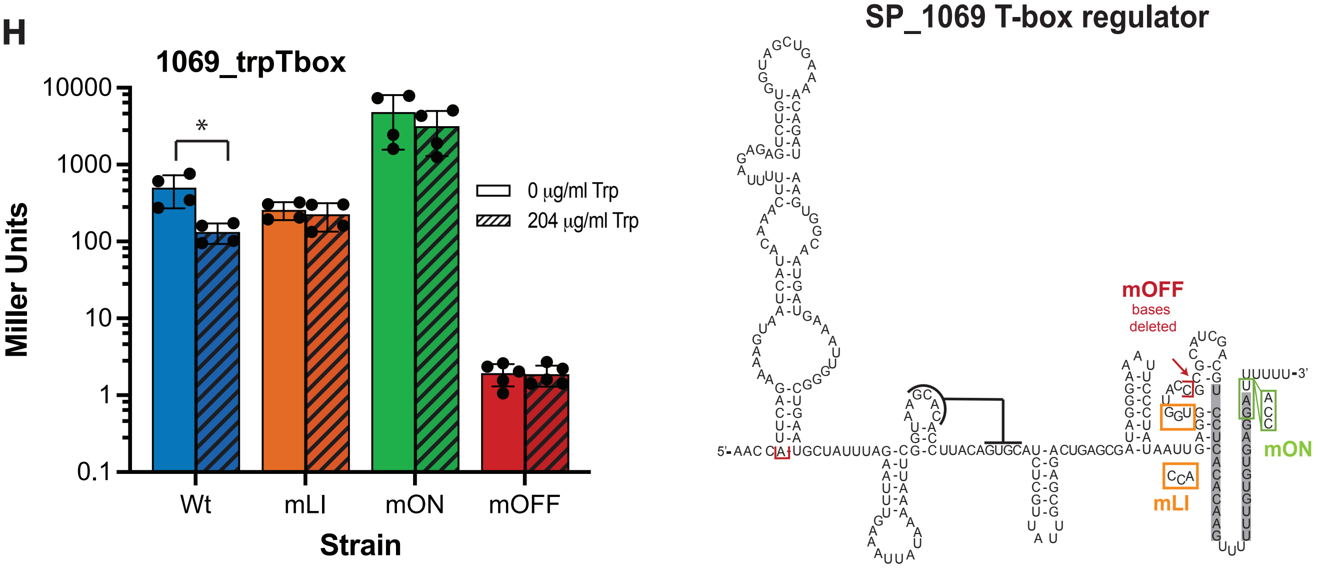

I

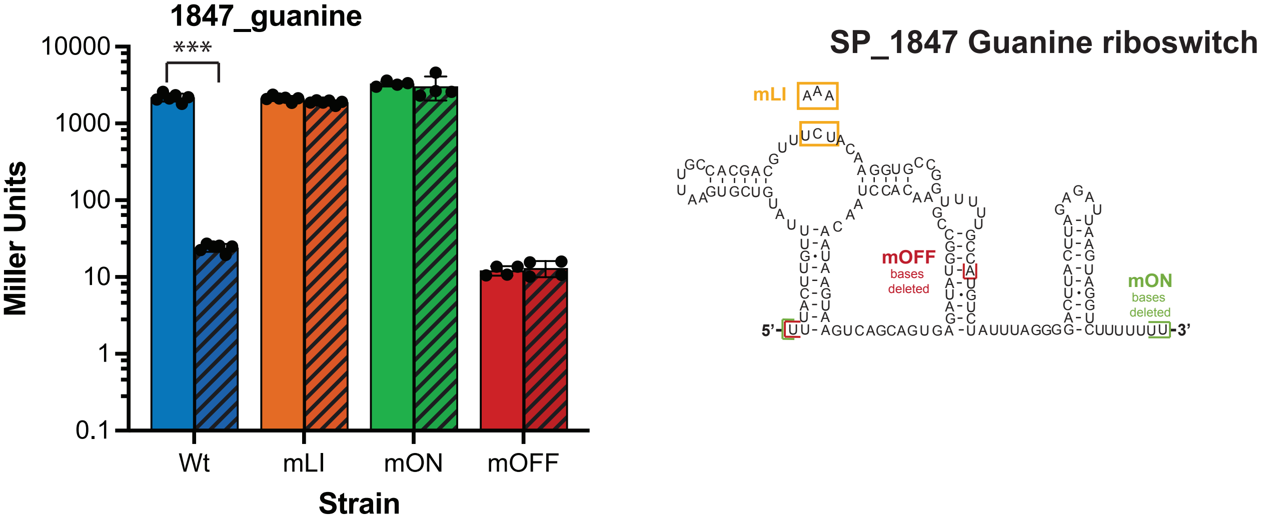

Figure S2 pg 4

J

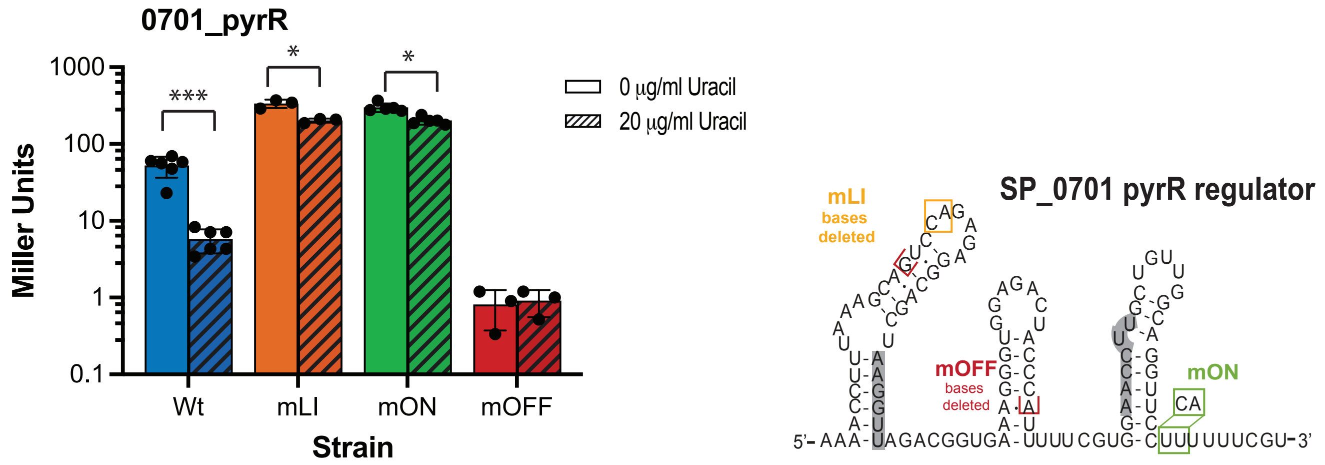

K

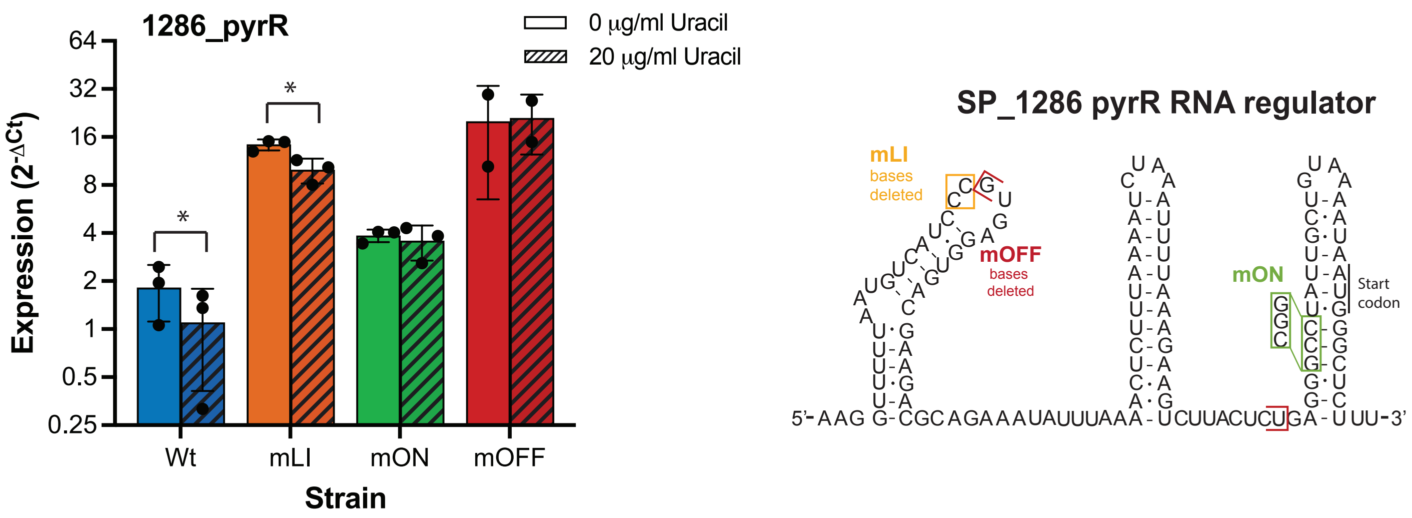

Supplement: S2 Fig — (A-K): Putative secondary structures are derived from aptamer consensus folding [65] and minimum free energy calculations [97]. Mutations are indicated on each structure. Some mutations are large deletions where the deleted nucleotides are bracketed by an appropriate color bracket (Ligand Insensitive = yellow, ON = green, OFF = red). β-galactosidase activity measured via Miller assay [94], and error bars represent standard deviation and individual biological replicates are indicated by points. Significance of mutant changes in activity upon ligand binding determined via one-way ANOVA followed by Sidak’s multiple comparisons test to compare values in the + and–ligand conditions for each mutant. (*p<0.05, **p<0.01, ***p < .001). WT samples are duplicated from Fig 1A for reference. (A-D) TPP riboswitch structures and mutants’ β -galactosidase activity in CDM lacking thiamine and including thiamine (-/+ 0.3 μg/ml thiamine). (E, F) FMN riboswitch structures and mutants’ β -galactosidase activity in CDM (-/+140 ng/mL riboflavin). (G) Glycine riboswitch structure and mutants’ β -galactosidase activity in CDM (-/+75 μg/mL glycine). % indicates values that are significantly different from one another, but not considered ligand responsive due to the direction of the response. (H) Tryptophan T-box structure and mutants’ β -galactosidase activity in CDM (-/+204 μg/mL tryptophan). (I) Guanine riboswitch structure and mutants’ β -galactosidase activity in CDM and CDM supplemented with 50 ug/mL guanine. (J) PyrR element preceding SP_0701 structure and mutants’ β-galactosidase activity in CDM (-/+ 20 μg/mL uracil). (K) PyrR element preceding SP_1286 and qRT-PCR measurement of gene expression. Individual points indicate biological replicates (average of 3 technical replicates). All numeric data points in S2 Data. (PDF) [file pgen.1011188.s009.pdf]
