# Supplementary material for: RNA cis-regulators are important for Streptococcus pneumoniae in vivo success
Source: PLoS Genet. 2024 Mar 5;20(3):e1011188. doi: 10.1371/journal.pgen.1011188 (PMC10942264; doi:10.1371/journal.pgen.1011188)
Supplement: Attachment — Submitted filename: PLOS-Gen-review-01.pdf [file pgen.1011188.s018.pdf]

RNA-based regulators are important elements that regulate genes in bacteria through direct ligand sensing (riboswitches), or recruitment of proteins or RNAs to sense the levels of metabolites in cells, thereby establishing cellular homoeostasis. However, studies of these elements rarely — if at all — examine how mutations to these elements influence bacterial fitness *in vivo*. Here, Warrier et al. examined fitness conferred by 12 regulatory *cis*-acting RNAs including multiple riboswitches, purR elements, and T-box motifs. Mutants of native loci were examined for each RNA that led to constitutively active or repressed genes. The authors found that growth defects in planktonic cultures of *S. pneumoniae* grown in vitro are associated with constitutive repression, whereas constitutive activation of gene expression was rarely deleterious. By contrast, an infectious disease model that assessed mouse nasal carriage and pneumonia using the same opportunistic pathogen indicated that both classes of mutations — constitutively active or constitutively repressed — reduced bacterial fitness. A challenging aspect of the work was the difficulty of conducting high-throughput analysis to evaluate the effects of disrupting a specific gene-regulatory RNA. Key findings include: (i) constitutive repression is deleterious for growth in media lacking target nutrients; (ii) constitutive activation and repression of gene expression decrease fitness during mouse infection; (iii) repression of gene expression is deleterious in planktonic culture, but mis-regulation is deleterious in vivo; (iv) S. pneumoniae is sensitive to repression of FMN biosynthesis and transport; and (v) S. pneumoniae is sensitive to mis-regulation of pyrimidine synthesis. Overall, this is an outstanding study and represents a landmark in the investigation of cis-acting RNA-mediated gene-regulatory elements in vivo. The work is data-rich and is extremely well written. The evidence is rooted in multiple replicates supported by controls and statistical analysis. The results provide a new rationale to target regulatory RNAs because in many cases proper regulation is just beneficial to the bacteria, but it is critical to mount a successful infection. The authors also conclude that metabolic control by cis-acting regulatory RNAs is fine-tuned in vivo, providing the means to spur evolution of various RNA regulatory mechanisms. Prior to publication, the authors must address a few concerns regarding the clarity of the presentation for the reader.

Clarifications

Line 244. Please explain the following statement further. “We also generated a control strain that integrates the Chlr cassette at an equivalent position with no changes to the regulator sequence (WTc).” In particular, what is meant by an “equivalent position”.

Line 291, how is the fitness value, W, calculated?

Fig. 4. How were the percentage values calculated in this figure?

Figure S4. For consistency and clarity, shouldn’t the y-axis be labeled “relative” fitness for panels a-c?

Figure S5e. The PyrR mOFF panel was excluded here. Please explain why this was omitted.
Fig 3b, lines 1110-1111. The authors make a strong case for loss of fitness in vivo when compared to the WT control. However, the bar graph in Figure 3b (blood) suggests there is a n

increase in fitness for mLI mutants over WT (teal bar in key), which is not described (lines 307-324) but is interesting and notable. Are the average fitness values in Fig. 3b statistically significant changes compared to WT? Some discussion and explanation of this observation is needed.

Fig. 5a. The legend of this figure is a little unclear about what is being compared in the upper box, and what is being reported in the lower blue boxes. The reader is forced to refer back to the supporting information to follow the flow and discern the meaning.

Lines 423-424. Could the authors describe what "cheating" behavior is for the co-culture studies.

Table S3. For consistency, the authors should label M1-M3, as mLI, mON, and mOFF, which will make the Table easier to read since the M1-M3 designations are infrequently used throughout the text. Fig. 5 also uses M3 designations.

Fig. 6d. The reviewer interprets the downward trend of OD 600 nm versus time as evidence of autolysis. Is this correct? If, so please add a brief statement to the text describing this for the reader, and the observation that it is not consistently observed in all KO cultures.

Fig. 7a. The authors noted that SP_1278, which corresponds to the PyrR regulatory protein, is very sensitive to dysregulation and shows many deleterious phenotypes in vitro. For clarity and to emphasis its importance, it should be added to the schematic diagram in panel 7a.

Typos

The authors should ascertain that they actually defined M1, M2 and M3. The reviewer recommends replacing these designations with mLI, mON, and mOFF.

Line 40 "development [of]"

Line 219. "many of the mutants show activity that [is] unresponsive to ligand".

Line 417, "similar to [the] way its growth".

Line 528, Fig S7G is just Fig. 7G.

Line 547, "may be due [to] differences in"

Line 732, insert a space "1[]ml PBS"

Line 809, "Daniel Beringer for [careful] copyediting of the manuscript."

Line 1224. (A-J) should be (A-K)

Line 1227, deleted “bases” should be deleted “nucleotides”

Line 1242. Add a full stop “uracil)”[.] (K)”

Line 1285, “of riboswitches interacting with the same ligand.” Should be “of cis-acting regulators interacting with the same ligand” because pyrR is not a riboswitch.

Line 1300, “[to] planktonic growth parameters”
