# Supplementary material for: RNA cis-regulators are important for Streptococcus pneumoniae in vivo success
Source: PLoS Genet. 2024 Mar 5;20(3):e1011188. doi: 10.1371/journal.pgen.1011188 (PMC10942264; doi:10.1371/journal.pgen.1011188)
Supplement: Attachment — Submitted filename: PLOS_Reviews_Rebuttal_013124.docx [file pgen.1011188.s019.docx]

Dear Editor,

Thank you for your prompt handing of our manuscript, the exceptionally fast turn-around especially during a time of the year when many are on holiday was much appreciated. We thank the reviewers for their help in improving the manuscript and we have addressed each reviewer’s comments below.

We hope that you will find the revised manuscript appropriate for publication in PLoS Genetics.

Sincerely,

Michelle M. Meyer, Ph.D.

Professor and Associate Chair of Biology

Boston College

Chestnut Hill, MA 02445

m.meyer@bc.edu

Reviewer #1: Summary:

In the manuscript entitled "RNA cis-regulators are important for Streptococcus pneumoniae in vivo success" submitted to PLOS genetics, Warrier and colleagues investigated how altering the functionality of 11 different riboswitches that respond to particular metabolites impact S. pneumoniae growth in chemically defined medium (CDM) and fitness in the blood, lungs, and nasopharynx of mice. Briefly, the authors mutate riboswitches that sense riboflavin, uracil, glycine, TPP, guanine, and charged tryptophan tRNAs levels so that they are no longer or less responsive to the metabolite, locked in an on-state, or always off. The authors first validate that the mutations have their intended effect, which do so in most cases (Figs. 1 and S2). The authors then determine the growth rate and yield for these strains in CDM (Figs. 2, S3, and S4), and fitness in the lungs, blood, and nasopharynx of mice (Figs. 3, S4, and S5) compared to a strain with the wild type riboswitch preserved. Overall, the authors find collectively that locking these riboswitches in the off position through mutation leads to S. pneumoniae growth defects in CDM particularly in the absence of its cognate metabolite, but the locked-off mutations as well as the less responsive and locked on mutations tend to cause a more substantial defect in S. pneumoniae fitness in vivo than in vitro (Figs. 2, 3, and 4). Overall, the authors find that disrupting riboswitches that sense uracil or riboflavin levels tend to have a more substantial effect on in vitro growth and in vivo fitness (Figs. 6 and 7), but find relatively poor correlation between the effects of mutations on growth rate or yield and in vivo fitness (Fig. 5).

In my opinion, the authors have done an impressive amount of work to establish the contribution of metabolite sensing riboswitches to the growth and fitness of S. pneumoniae. Moreover, they have demonstrated that gene regulation by riboswitches has a much more profound impact on the ability of S. pneumoniae to survive and thrive in the in vivo environments tested than in the vitro conditions examined. I have no substantial criticisms to offer regarding this work. I do have some suggested corrections to typographical errors that I found.

We thank the reviewer for their appreciation of our work.

Major Criticisms:

None.

Minor Concerns:

L19. Change to "a rapid response" *-done*

L40. Change to "development of" *-done*

L189. Change to "We found that" *-done*

Figure 2D and 2E. For clarity, the authors should add the term "Relative" to "Carrying Capacity" or "Growth Rate" at the top of the graph. *-done*

L411. Change to "observed" *-done*

L463. (Fig. S3L) is incorrectly referenced here. *– done change to S3M*

L508. FigS3L should be referenced along with Fig. S7B. – *Done.*

L528 Change to "Fig. 7G". also reference Fig. S3L. – *Done.*

We thank the reviewer for identifying these typos. We have corrected them in the manuscript.

Reviewer #2: The review was uploaded as an attachment. (Copied below)

RNA-based regulators are important elements that regulate genes in bacteria through direct

ligand sensing (riboswitches), or recruitment of proteins or RNAs to sense the levels of

metabolites in cells, thereby establishing cellular homoeostasis. However, studies of these

elements rarely — if at all — examine how mutations to these elements influence bacterial

fitness in vivo. Here, Warrier et al. examined fitness conferred by 12 regulatory cis-acBng RNAs

including multiple riboswitches, purR elements, and T-box motifs. Mutants of native loci were

examined for each RNA that led to constitutively active or repressed genes. The authors found

that growth defects in planktonic cultures of S. pneumoniae grown in vitro are associated with

constitutive repression, whereas constitutive activation of gene expression was rarely

deleterious. By contrast, an infectious disease model that assessed mouse nasal carriage and

pneumonia using the same opportunistic pathogen indicated that both classes of mutations —

constitutively active or constitutively repressed — reduced bacterial fitness. A challenging

aspect of the work was the difficulty of conducting high-throughput analysis to evaluate the

effects of disrupting a specific gene-regulatory RNA. Key findings include: (i) constitutive

repression is deleterious for growth in media lacking target nutrients; (ii) constitutive activation

and repression of gene expression decrease fitness during mouse infection; (iii) repression of

gene expression is deleterious in planktonic culture, but mis-regulation is deleterious in vivo;

(iv) S. pneumoniae is sensitive to repression of FMN biosynthesis and transport; and (v) S.

pneumoniae is sensitive to mis-regulation of pyrimidine synthesis. Overall, this is an outstanding study and represents a landmark in the investigation of cis-acting RNA-mediated gene-regulatory elements in vivo. The work is data-rich and is extremely well written. The evidence is rooted in multiple replicates supported by controls and statistical analysis. The results provide a new rationale to target regulatory RNAs because in many cases proper regulation is just beneficial to the bacteria, but it is critical to mount a successful infection. The authors also conclude that metabolic control by cis-acting regulatory RNAs is fine-tuned in vivo, providing the means to spur evolution of various RNA regulatory mechanisms. Prior to publication, the authors must address a few concerns regarding the clarity of the presentation for the reader.

Clarifications

Line 244. Please explain the following statement further. “We also generated a control strain

that integrates the Chlr casseTe at an equivalent position with no changes to the regulator

sequence (WTc).” In particular, what is meant by an “equivalent position”.

All of our mutant strains are created via homologous recombination and successful recombinants selected with a Chl^R^ marker. The WT^c^ strain has an equivalent ChlR marker, but no additional mutations to the regulatory sequence. We have clarified this in the text:

*“To assess the effect of RNA regulation on S. pneumoniae fitness we created a set of strains for each regulator where the native locus is replaced with each mutant (mLI, mON, and mOFF). To enable selection of the mutants following homologous recombination a Chl^r^ cassette is inserted upstream of the native promoter (Fig. 2A). We also generated a matched control strain for each regulator containing only the Chl^r^ cassette and no changes to regulatory sequence (WT^c^).”*

Line 291, how is the fitness value, W, calculated?

We define fitness fully in the methods, however to clarify in the context of the main text we have modified this section to give a better idea of what goes into the calculation quantifies, specifically reference the methods section, and ultimately cite this calculation more appropriately.

*“To determine the relative fitness of RNA regulator mutations during infection, we performed 1:1 competition assays between the parental strain (unmodified S. pneumoniae TIGR4) and each mutant in two mouse models: a nasopharynx colonization assay mimicking S. pneumoniae colonization and carriage where nasopharyngeal lavage is collected at 24-48 hours after infection, and a lung infection assay mimicking the S. pneumoniae invasive disease of pneumonia where the lung is collected 6-24 hours after infection. To assess vascular invasion following lung infection, blood was also collected from animals following the lung infection assay. From the initial inoculum and each collected sample, mutant (Chl^R^ mutant strain) and total CFUs (mutant and parental strain) were enumerated to allow calculation of the relative frequencies of each strain at both the start and end of the experiment. Thus, the fitness (W) of the mutant strain relative to the reference strain can be determined (see methods)(78).”*

In the methods, we give a more comprehensive description including a discussion of the equation used and the parameters that go into this equation.

Fig. 4. How were the percentage values calculated in this figure?

We have revised the legend to Fig. 4 to clarify the meaning of these percentages.

*“(****A****) Aggregated and sampled relative carrying capacity across all regulators (Fig. S3). A randomly chosen sampled population is depicted. A total of 100 random samplings of the data were performed and the percentage of sampled populations displaying a statistically significant difference between matched control constructs (WT^C^) and mutant strains is indicated (Kruskal-Wallis followed by Dunn’s test of multiple comparison, number of samplings/100 samplings where adj. p<0.05). Average p-values and percentage of sampled populations where p <0.05 reported in Table S5.”*

Figure S4. For consistency and clarity, shouldn’t the y-axis be labeled “relative” fitness for panels a-c?

We have corrected these figure labels.

Figure S5e. The PyrR mOFF panel was excluded here. Please explain why this was omitted.

A mutant for 1286_PyrR with constitutively repressed activity could not be generated (see Fig. 1G, exploded pie slice). Thus, we omitted this mutant from further studies. We have made a note in the legend to clarify this omission.

Legend to Fig. S5:

“**(E)** #1286_pyrR WT^C^, mLI, and mON (1286_pyrR_mOFF mutant not successfully constructed).”

Fig 3b, lines 1110-1111. The authors make a strong case for loss of fitness in vivo when compared to the WT control. However, the bar graph in Figure 3b (blood) suggests there is an

increase in fitness for mLI mutants over WT (teal bar in key), which is not described (lines 307-

324) but is interesting and notable. Are the average fitness values in Fig. 3b statistically

significant changes compared to WT? Some discussion and explanation of this observation is

needed.

There is one mLI where the average fitness in mice is >1.1. The average fitness value here it is 1.11 +/- 0.063 and not statistically significant from the WT^C^ value for 0178_FMN, which is 1.06. We now discuss this instance in the text.

*“While one strain (0178_FMN_mLI) does show W=1.1 in the lung (Fig. 3B, lung, Table S4), this finding is not statistically significant compared to the in vitro control (W=1.06).”*

Fig. 5a. The legend of this figure is a little unclear about what is being compared in the upper

box, and what is being reported in the lower blue boxes. The reader is forced to refer back to the supporting information to follow the flow and discern the meaning.

We revised the portion of the manuscript describing these results to increase clarity and flow through the manuscript. Since the chart in Fig. 5A is summarizes information also on graphs in the supplement (Fig. S7), we removed this chart, referenced the specific R^2^ values necessary for comparison in the text and refer to the graphs in Fig. S7, each of which indicate the R^2^ and adj. p-values for the appropriate comparisons. Thus, Fig. 5B is now Fig. 5A.

*“By directly comparing our modelled and measured fitness values, we find the strongest correlation is between modelled exponential fitness (approximately equal to the relative growth rate) and measured fitness in the lung (Fig. 5A, Fig, S7A-F). While significant, this correlation (R^2^=0.59) is weak compared to the correlations between in vivo fitness values from different environments (Fig. S7G-I, R^2^=0.84, 0.88, and 0.95).”*

Lines 423-424. Could the authors describe what “cheating” behavior is for the co-culture

studies.

We have further elaborated on the potential “cheating” behavior:

*“However, the general trends observed from our experiment match those expected from the modelling and potentially suggest that co-culture fitness as we are measuring it is a less sensitive metric than parameters extracted from monoculture growth curves due to potential cheating behavior wherein the mutant strain, which cannot synthesize pyrimidines itself, obtains sufficient amounts from the co-cultured wild-type strain.”*

Table S3. For consistency, the authors should label M1-M3, as mLI, mON, and mOFF, which will make the Table easier to read since the M1-M3 designations are infrequently used throughout the text. Fig. 5 also uses M3 designations.

We have changed both of these references to M1, M2, and M3. The change in terminology was late in the writing process and we apologize for the confusion.

Fig. 6d. The reviewer interprets the downward trend of OD 600 nm versus time as evidence of

autolysis. Is this correct? If, so please add a brief statement to the text describing this for the

reader, and the observation that it is not consistently observed in all KO cultures.

The reviewer is correct, this is autolysis. We do not routinely observe autolysis in CDM, but do SDMM. We have clarified our description of autolysis in the main text to draw the contrast with what we typically observe in CDM.

*“However, one aspect of the growth curves not captured by our quantitative analysis is the autolysis of S. pneumoniae, which results in a decline in culture density (OD_600_) following exponential growth. Autolysis is characteristic for S. pneumoniae TIGR4 grown in rich medium (Fig. S3M), but often not observed in CDM (Fig. S3, Fig. S6D). The SP_0178_KO strain appears to undergo autolysis in CDM (Fig. 6D) whereas our other strains typically do not, or do so after a more extended plateau. This decline may indicate that this strain is under some additional stress that is not captured by our other measures.”*

Fig. 7a. The authors noted that SP_1278, which corresponds to the PyrR regulatory protein, is

very sensitive to dysregulation and shows many deleterious phenotypes in vitro. For clarity and to emphasis its importance, it should be added to the schematic diagram in panel 7a.

We have added SP_1278 (PyrR) to our diagram in Fig. 7a and modified the legend appropriately.

Typos

The authors should ascertain that they actually defined M1, M2 and M3. The reviewer

recommends replacing these designations with mLI, mON, and mOFF.

We have replaced any remaining M1, M2, M3 designations with mLI, mON, and mOFF.

Line 40 “development [of]”

Line 219. “many of the mutants show activity that [is] unresponsive to ligand”.

Line 417, “similar to [the] way its growth”.

Line 528, Fig S7G is just Fig. 7G. -

Line 547, “may be due [to] differences in”

Line 732, insert a space “1[]ml PBS” -

Line 809, “Daniel Beringer for [careful] copyediting of the manuscript.”

Line 1224. (A-J) should be (A-K)

Line 1227, deleted “bases” should be deleted “nucleotides”

Line 1242. Add a full stop “uracil)”[.] (K)”

Line 1285, “of riboswitches interacting with the same ligand.” Should be “of cis-acting regulators interacting with the same ligand” because pyrR is not a riboswitch.

Line 1300, “[to] planktonic growth parameters”

We thank the reviewer for their patience with our typos and we have corrected them.

Reviewer #3: In their study, Warrier et al analyzed the impact of 12 highly conserved RNA cis-regulators inhibited in presence of various metabolites in in vitro and in vivo growth of Streptococcus pneumoniae. They introduced specific mutations either to perturb ligands binding (mLI), or to constitutively activate (mON) and repress (mOFF) expression. The key result was that miss-regulation (positive or negative) induced a stronger fitness decrease in mouse infection than in growth medium for 75% of the mutant strains. The authors emphasized their findings with two specific examples: FMN and uracil responsive RNA elements. In brief, the work was original, the experiments were well conducted, and the results were clearly presented. Below are few comments and suggestions:

We appreciate the reviewer’s positive reception of our work.

Because all the riboswitches were repressed in presence of ligands (Fig 1A) and mLI/mON mutations presented a greater activity than the WT in vitro (Fig 1D and comment below), it is difficult to assume whether the RNA regulators were ON or OFF in vivo. I would suggest that the authors check the relative expression of one of the WT RNA regulators, and its derivatives in organs by qRT-PCR. Furthermore, the authors should discuss the apparent paradox that the mON and mOFF mutants behaved similarly in infection models.

We agree with the reviewer, that it is difficult to assume what the behavior of the regulated genes may be *in vivo*. In particular, the genes may not be expressed at all due to promoter level effects. We do believe that our mutants likely behave similarly, within the layered context of gene control. For example, if the gene is transcribed, our ON mutant will be ON more than the WT.

However, the experiment described is exceedingly technically challenging. Typically, only 10^5^-10^8^ CFU are recovered total from each tissue during the *in vivo* environments, and these cells are mixed with a substantial amount of mouse tissue. There is also no obvious method to enrich bacterial RNA outside the amplification event itself. This is one of the reasons that learning about bacterial physiology *in vivo* via dual RNA-seq experiments has been so challenging to date.

We did review the literature and identified a sequencing study of *S. pneumoniae* during infection (D’Mello et al. PNAS 2020). From the supplementary data published with this work, we observe that all of the RNA-regulated transcripts we assessed in mice are at least detectable (although some at very low levels) in one or more of the *in vivo* environments. Thus, we argue that our mutants likely behave in a way reflected by the in vitro Miller assays.

We added sections to the discussion to address this ambiguity in our work as well as the apparent paradox that the mON and mOFF mutants both result in fitness decreases in vivo.

*“That both constitutive repression (mOFF) and constitutive activation (mON) lead to decreases in fitness may be considered somewhat paradoxical. However, in vivo fitness is a property that encompasses many different characteristics including survival in an adverse environment, successful attachment to and invasion of host cells, and ultimately replication. As such, under- and over-expression may impact different characteristics, yet ultimately yield similar impacts on fitness.” …*

*“In addition, while we have assayed the gene-expression of our mutants in culture, we do not have direct evidence of the gene-expression during infection. In particular, the regulatory mechanisms we assess here are all post transcription initiation, thus the genes must be transcribed before mutations have a functional impact. Our findings suggest that the transcripts are produced in vivo, as at least one mutant of each regulator displayed a statistically significant fitness deficit in one or more in vivo conditions. In addition, assessment of existing dual RNA-seq data from TIGR4 in these environments indicates that the studied transcripts are detectable during infection (90), although the extent of expression varies widely. Notably, SP_0488, whose expression we found to be critical for viability during infection is the most highly expressed of these transcripts in vivo (Table S6). Thus, we anticipate that our mutants with constitutive activation or repression likely behave similarly in vivo as they do in vitro with similarly increased or decreased expression. However, the caveat that there may be additional regulatory mechanisms acting that are not apparent in vitro remains.”*

L674: “50S ribosomal protein gene”, change to “L33 50S ribosomal protein gene”
L221-224: what are the authors hypotheses about the increase expression of the mON and mLI mutants? This should be discussed.

We have added speculation regarding the mechanism for increased expression of the mON and mLI mutants compared to the unrepressed WT.

*“For both the mLi and mON mutants the increased gene expression observed is likely due to stabilization of the transcript in a form where read-through of the intrinsic terminator is favored. We, and others, have observed in 3’-sequencing studies that many riboswitches alter the extent of read-through for intrinsic terminator stems, and it is rarely 0% or 100% utilization for such conditional terminators (7,64). Thus, it is entirely possible that our mutation may stabilize an anti-terminator stem to a greater extent than it would be in the absence of ligand of a wild-type strain. Furthermore, many mON mutants completely remove the intrinsic terminator. We speculate that the retained ligand responsiveness may derive from promoter level regulation of transcription initiation, which is unperturbed in our mutants.”*

From L506: the paragraph is very confusing. The authors referred to in vivo results for the 1278_pyrR-mOFF mutant that I could not find. The legend of Figure S7 is not clear: to which RNA regulator does it refer to?

The reference to S7 in this paragraph was a typo, referring to Fig. 7, this has been corrected. The 1278_pyr-mOFF mutants are previously published, and in the interest of transparency we omitted directly repeating any of the previously published data in this manuscript, although we did report the quantified growth parameters from those growth curves (Fig, 3L) as these had not previously been reported. We have modified the legend to Fig. 7 to make it clearer that this is existing previously published data from our group that is available in another publication.

*“Data corresponding to growth curves and mouse infections for mutants of SP_1278 is previously published (64).”*
